# On the potential roles of phosphorus in the early evolution of energy metabolism

**DOI:** 10.3389/fmicb.2023.1239189

**Published:** 2023-08-02

**Authors:** Jack W. F. Nicholls, Jason P. Chin, Tom A. Williams, Timothy M. Lenton, Vincent O’Flaherty, John W. McGrath

**Affiliations:** ^1^School of Biological Sciences, Queen’s University of Belfast, Belfast, United Kingdom; ^2^School of Biological Sciences, University of Bristol, Bristol, United Kingdom; ^3^Global Systems Institute, University of Exeter, Exeter, United Kingdom; ^4^School of Natural Sciences, University of Galway, Galway, Ireland

**Keywords:** origins of life, primordial metabolism, adenosine triphosphate (ATP), bioenergetics, last universal common ancestor (LUCA), polyphosphate, acetyl phosphate, phosphite

## Abstract

Energy metabolism in extant life is centered around phosphate and the energy-dense phosphoanhydride bonds of adenosine triphosphate (ATP), a deeply conserved and ancient bioenergetic system. Yet, ATP synthesis relies on numerous complex enzymes and has an autocatalytic requirement for ATP itself. This implies the existence of evolutionarily simpler bioenergetic pathways and potentially primordial alternatives to ATP. The centrality of phosphate in modern bioenergetics, coupled with the energetic properties of phosphorylated compounds, may suggest that primordial precursors to ATP also utilized phosphate in compounds such as pyrophosphate, acetyl phosphate and polyphosphate. However, bioavailable phosphate may have been notably scarce on the early Earth, raising doubts about the roles that phosphorylated molecules might have played in the early evolution of life. A largely overlooked phosphorus redox cycle on the ancient Earth might have provided phosphorus and energy, with reduced phosphorus compounds potentially playing a key role in the early evolution of energy metabolism. Here, we speculate on the biological phosphorus compounds that may have acted as primordial energy currencies, sources of environmental energy, or sources of phosphorus for the synthesis of phosphorylated energy currencies. This review encompasses discussions on the evolutionary history of modern bioenergetics, and specifically those pathways with primordial relevance, and the geochemistry of bioavailable phosphorus on the ancient Earth. We highlight the importance of phosphorus, not only in the form of phosphate, to early biology and suggest future directions of study that may improve our understanding of the early evolution of bioenergetics.

## 1. Introduction

Phosphorus, usually in the form of inorganic orthophosphate (phosphate, PO_4_^3−^), is central to the biochemistry of all life in the storage of genetic information (RNA and DNA), structure of cellular membranes, phosphorylation of metabolites and regulation of enzymes. Phosphorylated nucleotides, principally adenosine triphosphate (ATP), are relied upon for the storage, transfer, and release of cellular energy, driving unfavorable metabolic reactions and phosphorylating metabolites. ATP is uniquely suited to its role as the ‘universal energy currency’ due to the highly energetic phosphoanhydride bonds (O=P-O-P=O) linking the orthophosphate anions of the triphosphate group (Westheimer, 1987). The polyanionic nature of the triphosphate group also stabilizes the nucleotide under varying physiological conditions, allowing enzymes to direct metabolic processes in specific energetic directions *via* hydrolysis or phosphorylation (Liu et al., 2019).

Given that all organisms use ATP as the major cellular energy currency, the centrality of phosphorylated nucleotides to energy metabolism must have been established before the divergence of the major domains of life at least 4 billion years ago (Cornish-Bowden and Cárdenas, 2017; Betts et al., 2018; Mahendrarajah et al., 2023). ATP is synthesized *via* substrate-level phosphorylation (SLP) or chemiosmotic coupling, both mechanisms are universally conserved and found in the last universal common ancestor (LUCA) of all domains of life. However, SLP requires numerous complex enzymes for the hydrolysis and phosphorylation of metabolic intermediates, while chemiosmosis requires ionic membrane gradients combined with a seemingly highly evolved ATP synthase molecular motor. Additionally, while LUCA is ancient, it was still a cellular organism with a genetic code and proteins. Thus, both LUCA and ATP-based bioenergetics were clearly the product of significant prior evolution (Gogarten and Deamer, 2016). Modern energy metabolism also has an autocatalytic requirement for ATP, either to produce the purine nucleotides required for *de novo* ATP synthesis or for the regeneration of ATP from its hydrolysis products adenosine mono−/di-phosphate (AMP/ADP) (Bonora et al., 2012; Pinna et al., 2022). Therefore, the relative complexity of ATP-based bioenergetics and the prerequisite for phosphorylated nucleotides implies the existence of evolutionarily simpler bioenergetic pathways, potentially utilizing primordial precursors to ATP, or other energetic compounds unknown to or abandoned by most extant biology.

The reliance on phosphate in modern energy metabolism, along with the unique energetic properties of phosphorylated compounds and their potential roles in phosphorylating the primordial building blocks of life, suggests that phosphate played a vital role in the early evolution of metabolic systems (Gilbert, 1986; Schwartz, 2006; Fernández-García et al., 2017). It is, however, difficult to reconcile this view with the long-held assertion that biologically available phosphate was scarce on the ancient Earth (Gulick, 1955; Schwartz, 2006; Pasek, 2008). Our increasing understanding of the ancient Earth’s geochemistry, coupled with the expanding metabolic diversity of modern organisms, may provide clues as to how early life powered its metabolism with or without phosphate. Microorganisms, sometimes living in extreme environments potentially analogous to the primordial Earth, have evolved diverse mechanisms to scavenge energy and phosphate from a variety of phosphorus compounds that might have primordial relevance, potentially as descendants of ancient bioenergetic or phosphate-scavenging pathways.

Herein, we will discuss and compare the various bioenergetic phosphorus compounds that could have been involved in primordial energy metabolism either as environmental sources of energy and phosphate, or primordial energy currencies pre-dating ATP-based bioenergetics entirely. The possible geochemical and biochemical routes that life may have taken to attain these compounds will be reviewed, along with the evidence for such metabolic processes that might exist in modern organisms. The potential for reduced phosphorus species to have provided primordial life with a unique source bioavailable phosphorus and energy through an ancient phosphorus redox cycle will also be highlighted.

## 2. Energy in ancient life

**Figure 1:**
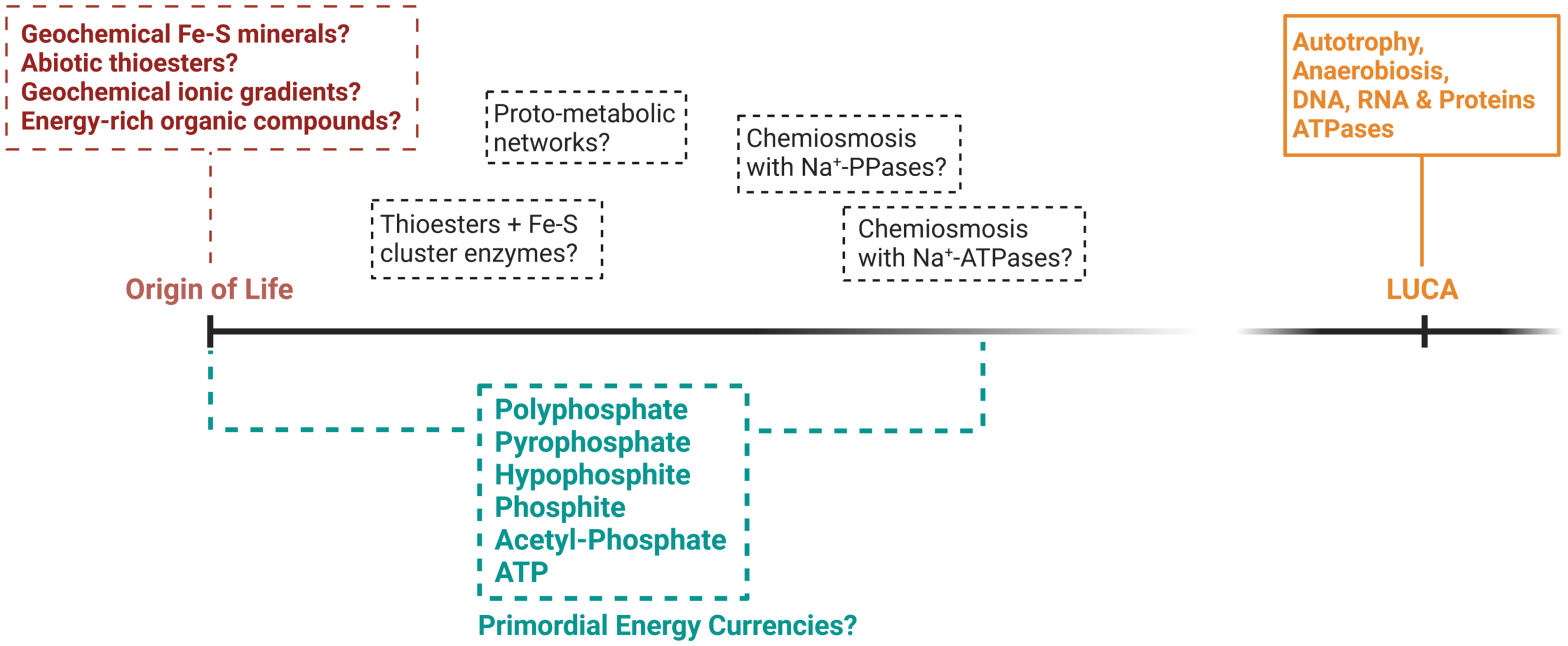
A speculative representation of the potential theories surrounding the origin and early evolution of energy metabolism. Included are some of the bioenergetic phosphorus compounds that might have provided energy for life during its origin and up to the time of LUCA. While LUCA is largely recognized as metabolically similar to modern prokaryotes, what came before LUCA is mostly hypothetical and indicated by dashed lines.

Many attempts have been made to explain how primordial life acquired energy prior to the evolution of the complex metabolism of LUCA (summarized in Figure 1), yet significant knowledge gaps remain. If we discount forms of energy that have left no trace in extant life, such as mechanical energy (Hansma, 2020) or UV phototrophy (Patel et al., 2015), the first organisms were probably chemotrophic. Chemoheterotrophy, involving the consumption of a primordial soup of organic and energetic compounds, is one of the oldest origin of life theories (De Duve, 1995; Follmann and Brownson, 2009). This soup of biomolecules might have formed *via* the prebiotic geochemistry of certain environments such as lakes, hot springs and rock pools, or might have been delivered to the early Earth *via* meteorites (Kitadai and Maruyama, 2018). However, the warm and anoxic environment of the prebiotic Earth rich in greenhouse gases might point more favorably to a chemoautotrophic origin of life, involving the synthesis of organic compounds from the fixation of inorganic carbon using energy derived from inorganic electron donors (Forterre and Gribaldo, 2007; Arndt and Nisbet, 2012; Schönheit et al., 2016; Catling and Zahnle, 2020).

Chemoautotrophs are ubiquitous in modern extreme environments, fixing inorganic CO_2_ and surviving on electrons and energy drawn from inorganic electron donors such as H_2_, Fe, NO_3_ or SO_4_. Environments similarly rich in inorganic minerals and an atmosphere of CO_2_ and ammonia were likely common on the ancient Earth (Sousa et al., 2018). Accordingly, chemoautotrophy has become one of the prevailing theories explaining how primordial life might have acquired energy. Chemoautotrophic carbon fixation *via* autocatalytic networks of small metabolites has often been proposed to represent the origin of metabolic life (Semenov et al., 2016; Martin and Thauer, 2017; Goldford et al., 2019; Preiner et al., 2020). Proto-metabolic networks may have been catalyzed by a variety of metal-containing minerals, such as iron, sulfur and copper, which were likely widespread on the prebiotic Earth and could have evolved into the oxidoreductase enzymes that are vital and ubiquitous in modern metabolism (Moore et al., 2017). Hydrothermal environments on the ancient Earth likely produced all the requirements of chemoautotrophy through the fixation of CO_2_ catalyzed by natural minerals (Preiner et al., 2018), and might also have provided energy in the form of geochemical ionic gradients coupled to primordial chemiosmotic systems (Lane, 2017).

Existing in an anaerobic world long before the great oxidation event, LUCA was also probably an anaerobic chemoautotroph. Consistent with this view is that genes involved in the fixation of inorganic carbon via the Wood-Ljungdahl (or reductive acetyl CoA) pathway and the reverse tricarboxylic acid (rTCA) cycle are consistently inferred in LUCA (Braakman and Smith, 2012; Weiss et al., 2016; Nunoura et al., 2018; Weiss et al., 2018; Coleman et al., 2021). Therefore, LUCA was probably synthesizing ATP via SLP in ancestral versions of carbon-fixation pathways and likely also contained an early chemiosmotic system, coupling natural or generated Na^+^ ion gradients to Na^+^-ATP synthases, instead of the H^+^ ion chemiosmotic coupling ubiquitous in modern organisms (Lane and Martin, 2012; Sousa et al., 2016; Weiss et al., 2016; Wimmer et al., 2021b). The rTCA and Wood-Ljungdahl pathways are utilized by many modern extremophilic and chemoautotrophic bacteria and archaea living in anoxic environments potentially analogous to the primordial Earth and, therefore, may represent a direct evolutionary link to simpler chemoautotrophic systems at the origin of life.

The evolutionary steps that occurred between the inferred metabolic traits of LUCA and the origin of life remain largely a mystery. What appears clear is that by the time of LUCA’s existence, life had evolved complex bioenergetic networks that relied heavily on phosphorylated molecules, synthesizing ATP *via* SLP and chemiosmotic coupling, utilizing ancestral versions of modern carbon fixation pathways and ATP synthases. While suggestions that biochemistry emerged from prebiotic geochemistry through the utilization of natural ionic gradients or minerals catalyzing primordial carbon fixation pathways at hydrothermal vent systems appear plausible, chemiosmotic gradients coupled to even primordial ATP synthases are likely too complex to represent the origin of bioenergetics (Jackson, 2016). Furthermore, modern SLP pathways also require evolutionary complex enzymes producing relatively small amounts of ATP and have an autocatalytic requirement for ATP and ancestral pathways might also suffer from these potential drawbacks of utilizing ATP. Whether and how primordial systems resembled modern metabolism in any way, and when recognizably modern metabolism first evolved, remains heavily debated. Therefore, considering alternatives to ATP in primordial energetic systems could provide insights that might circumvent some of the difficulties with primordial ATP-based bioenergetic systems.

## 3. Ancient life and the phosphate problem

Our understanding of the potential primordial roles of phosphorus, as sources of environmental energy or as constituents of ancestral energy currencies, is intrinsically linked to its bioavailability on the ancient Earth. While phosphorus is ubiquitous in the lithosphere today, existing primarily in the P^5+^ oxidation state as inorganic orthophosphate, the abundance of Ca^2+^ ions in the lithosphere results in its sequestration by insoluble and unreactive apatite minerals [Ca_5_(PO_4_)_3_(OH, F, Cl)] locked within igneous and metamorphic rock (Maciá, 2005). Apatite mineral formation *via* the precipitation and sedimentation of orthophosphate in the calcium-rich oceans is the major sink of biologically available phosphate today (Ruttenberg, 2003). Seafloor hydrothermal systems, where oceanic crust is made and fluids are exchanged, are also a net sink of phosphate from today’s oxidizing oceans. The principal input of bioavailable phosphorus comes in the form of soluble phosphate from the geological weathering and microbial solubilization of apatite minerals. The phosphate released *via* this route is minor compared to the global biological phosphorus demand, with most bioavailable phosphate sourced from the turnover of biomass. Thus, as the main environmental source of bioavailable phosphorus in today’s biosphere, the insolubility and mineral sequestration of most inorganic phosphate is a significant constraint on biological productivity in environments worldwide (Karl, 2014; Du et al., 2020).

While the bioavailability and abundance of phosphate on the early Earth remains a heavily debated subject (Pasek et al., 2017; Pasek, 2020; Walton et al., 2021, 2023), the early oceans were probably similarly rich in calcium as today and, therefore, likely caused widespread mineral sequestration of phosphate (Hazen, 2013). Additionally, iron oxides, delivered in abundance by volcanic activity and solubilized by the more reducing and CO_2_-rich early atmosphere, also sequestered free phosphate. This sequestration is illustrated in banded iron formations laid down in the ancient oceans (Bjerrum and Canfield, 2002), although to what extent remains debated (Brady et al., 2022). Weathering of terrestrial rock, a major source of bioavailable phosphate in today’s lithosphere, was also significantly reduced due to the absence of widespread microbial solubilization and the smaller surface areas of early granite continents and basaltic volcanic islands (Arndt and Nisbet, 2012). Sea-floor weathering was also an unlikely source of soluble phosphate (Mills et al., 2014). Additionally, the highly reduced phosphorus species phosphine (PH_3_), if present at all on the early Earth, would have been rapidly oxidized, even by the less oxidizing early atmosphere (Glindemann et al., 1999; Catling and Zahnle, 2020; Omran et al., 2021). Thus, the scarcity of soluble and bioavailable phosphate may have been amplified on the Hadean and early Archean Earth when life was first evolving.

**Figure 2:**
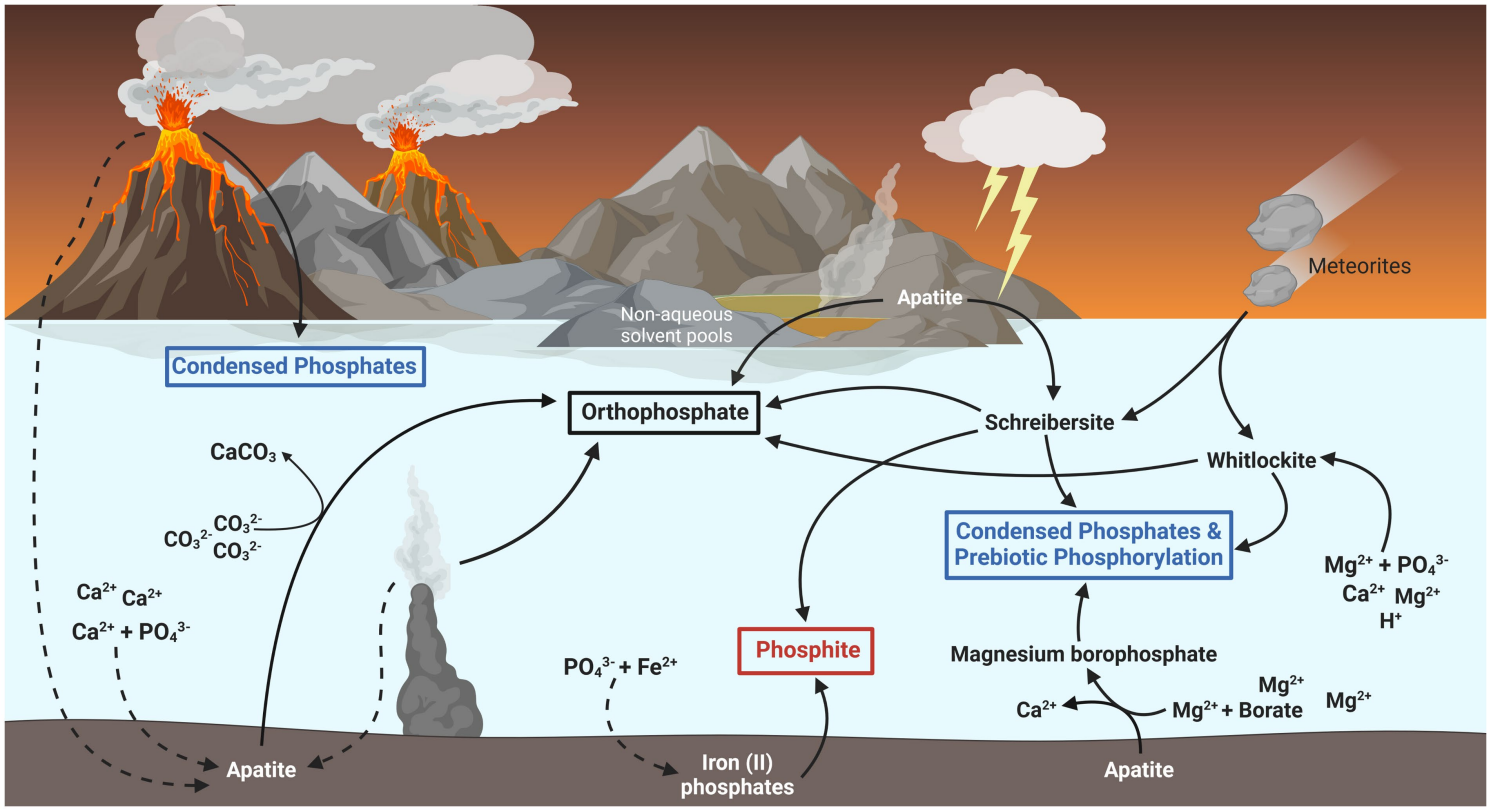
Potential fluxes of biologically relevant phosphorus on the early Earth. Most inorganic phosphate (PO_4_^3−^) on the late Hadean and early Archaean Earth was likely sequestered as the mineral apatite [Ca_5_(PO_4_)_3_(Fl, Cl, OH)], indicated by dashed lines. Biologically available forms of phosphorus might have existed in the form of schreibersite and whitlockite minerals, which dissolve into phosphite (red box) and phosphate (black box) respectively. These minerals have also been shown to form condensed phosphates (blue box), such as pyrophosphate and polyphosphates, and phosphorylate biomolecules essential for early life. Phosphite might also have formed through the adsorption and reduction of phosphate by iron (II) oxides likely found in the early ocean. Other sources of bioavailable phosphate may have existed as smaller fluxes from condensed phosphates formed in volcanic fumaroles, the liberation of phosphate from apatite by carbonate ions, and the dissolution of apatite in non-aqueous environments such as urea and ammonia pools or deep eutectic solvents.

In the last couple decades, several solutions to the problem of phosphate bioavailability on the ancient Earth have been posited (illustrated in Figure 2). Apatite, and other phosphate-containing minerals such as struvite, become significantly more soluble in such solvent environments and can facilitate the polymerization and phosphorylation of biomolecules without the potential thermodynamic instability involved with phosphorylated biomolecules in water (Gibard et al., 2018; Nascimento Vieira et al., 2020). Apatite minerals might have also released soluble phosphate if the early oceans were more acidic, hotter, and richer in magnesium than is normally predicted (Arndt and Nisbet, 2012; Pasek et al., 2017; Walton et al., 2021). Whitlockite is another more soluble phosphate mineral able to phosphorylate biomolecules and generate condensed phosphates (polyphosphates) upon heating (Keefe and Miller, 1995). Whitlockite might have precipitated out of a more acidic and magnesium-rich ancient ocean (Gedulin and Arrhenius, 1994) or could have been delivered as scarce components of meteorites (Adcock et al., 2017).

Another solution involves the replacement of water as the ubiquitous biological solvent. Evaporation pools, or other geochemically active areas experiencing wet and dry cycles, might have resulted in concentrated chemical solutions of urea, ammonia, formate and water (UAFW), or deep eutectic solvents consisting of salts and urea (Burcar et al., 2016, 2019; Lago et al., 2020). Other phosphate-solubilizing mechanisms have been proposed such as: the removal of phosphate-binding calcium by carbonate sequestration (Toner and Catling, 2020), the solubilization of phosphate from borate-containing evaporite deposits (Kim et al., 2016), and the precipitation of the slightly more soluble magnesium ammonium phosphate salts (i.e., struvite), in a preferential manner to apatite mineralization (Holm, 2012; Lago et al., 2020). Underwater hydrothermal vents could have also acted as source of phosphate able to avoid sequestration and supported by a more chemically reducing Archean ocean (Rasmussen et al., 2021).

However, many of these mechanisms are predicated on the existence of unique microenvironments on the early Earth and the existence of such environments remains uncertain. While urea and ammonia were undoubtably present on the early Earth, the existence of non-aqueous solvent environments is questionable and several plausible routes to proto-biochemistry exist in water (Gibard et al., 2018; Nascimento Vieira et al., 2020). The more soluble phosphate minerals, such as whitlockite and struvite, were also likely scarce and generally predicted to have had little relevance as abiotic sources of phosphorus (Hazen, 2013; Walton et al., 2021). Yet, while life may have plausibly begun in niche microenvironments consisting of unique prebiotic geochemistry, it soon spread across the Earth and would have required a regular source of soluble phosphorus likely significantly higher than that found in most current oceans (Schwartz, 2006). The chemical composition of the early ocean, while uncertain, is generally predicted to have been as similarly limited in magnesium and abundant in calcium as today (Jones et al., 2015). Furthermore, after life had taken hold, the sequestration and eventual sedimentation of phosphate in biomass would then become another major sink, as it is today. For a long-term sustainable system, there must have been a significant and regular source of phosphorus on the early Earth sufficient to counterbalance these sinks.

A recently characterized and increasingly plausible source of biologically available phosphorus appears to come from reduced phosphorus species, mainly in the form of phosphide (P^3−^) minerals like schreibersite [(Fe, Ni)_3_P]. As a constituent component of most meteorites, schreibersite would have been delivered in abundance during the late heavy bombardment in the Hadean period and has been found in Archaean rocks (Hazen, 2013; Bindi et al., 2023). While meteorites are a sporadic source, this could have been supplemented by schreibersite produced directly on the Earth’s surface *via* the action of lightning strikes on soil (Hess et al., 2021). Schreibersite produced this way may have represented a significant source of phosphorus, sustained even after the reduction of meteorite impacts after the late heavy bombardment. Phosphite originating from schreibersite may have even comprised as much as 5–10% of all world phosphorus during the late Hadean and Archaean (Hazen, 2013; Pasek et al., 2013).

Schreibersite has significance for primordial life due to its ability to dissolve under various conditions to produce various biologically available phosphorus compounds, including orthophosphate, polyphosphates and organic phosphates, under potentially prevalent conditions on the ancient Earth (Pasek and Lauretta, 2005; Bryant et al., 2013). Phosphide minerals are also able to completely oxidize to orthophosphate in prebiotic conditions through the action of UV light (Ritson et al., 2020). Additionally, schreibersite and its dissolution products can directly perform a variety of biologically relevant phosphorylations and polymerizations, including those of nucleotides and other organics (Gull et al., 2015; Pasek, 2017; Gibard et al., 2018). The dissolution of phosphide minerals into inorganic phosphate would appear to have resulted in the same problem as every other source of soluble phosphate, namely its sequestration by iron and calcium minerals. However, as will be discussed further in this review, phosphide minerals producing reduced phosphorus compounds under mild, and potentially primordial, conditions could have represented a significant source of soluble phosphorus for biomass, and even energy, during the early evolution of life.

## 4. Phosphorylated molecules in ancient energy metabolism

**Figure 3:**
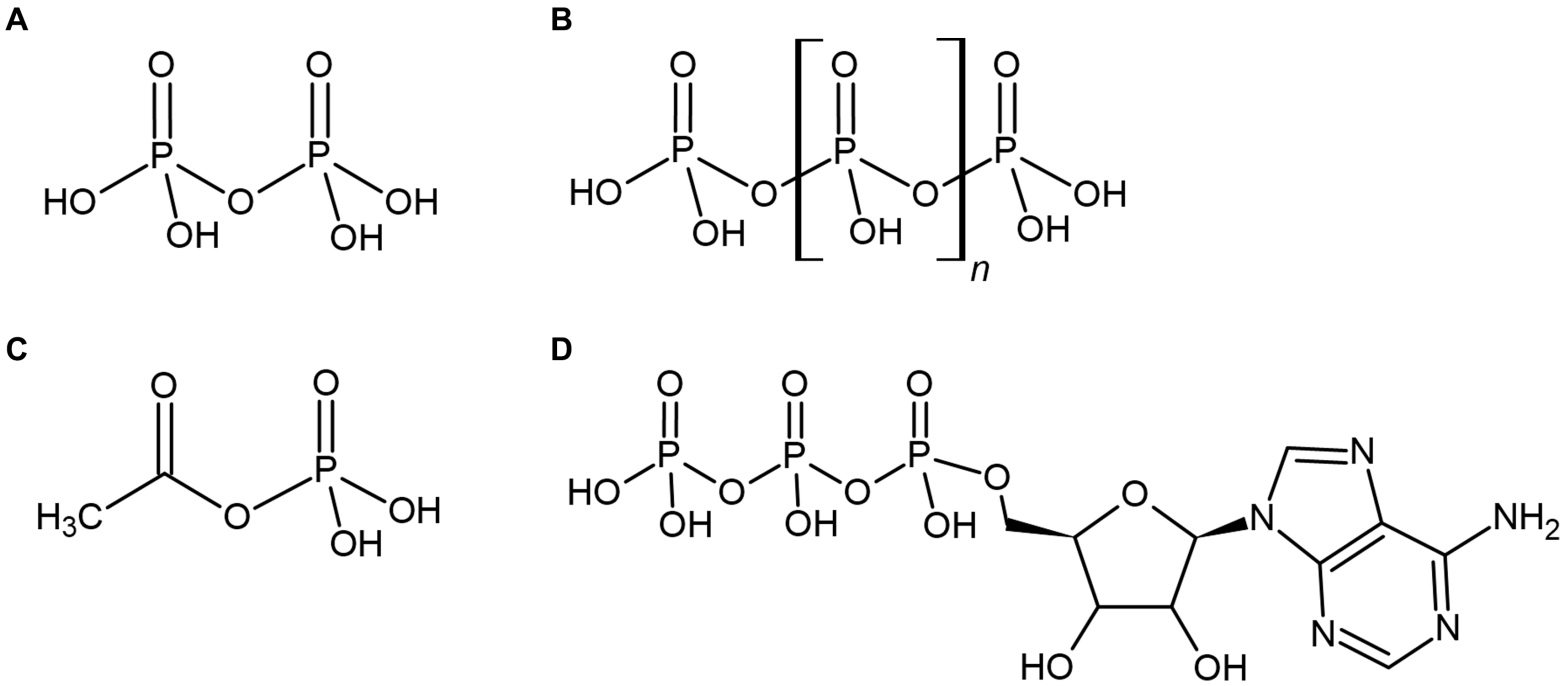
Various phosphorylated compounds could have been utilized by primordial life, such as the condensed orthophosphate esters pyrophosphate **(A)** and polyphosphate **(B)**, or the thioester-related compound acetyl phosphate **(C)**. These compounds are often suggested to pre-date the universal energy currency of extant life, adenosine triphosphate (ATP) **(D)** or have been utilized as environmental sources of biological energy.

If bioenergetic systems existed prior to the evolution of ATP-based metabolism, they may also have utilized the unique properties of phosphate and phosphorylated energy currencies. Consequently, various energetic phosphate-containing compounds become compelling intracellular energy currencies that could have been synthesized by primordial life prior to the adoption of ATP (Figures 3A–D). These highly energetic compounds may also have provided primordial life with a significant source of environmental energy or phosphate, where orthophosphate may have been severely limited. These various phosphorylated molecules and their related primordially relevant applications are considered below.

### 4.1. Pyrophosphate

Pyrophosphate, two orthophosphate residues bound by a phosphoanhydride bond (Figure 3A), has long been discussed as an alternative source of energy for microbes and as a potentially ancient cellular energy currency (Lipmann, 1965; Baltscheffsky et al., 1966; Peck et al., 1983) as it is a common byproduct of numerous biochemical reactions – including the hydrolysis of ATP, and the polymerization of DNA and proteins. The discovery, in the 1960’s, of membrane-bound pyrophosphatases (PPases) further reinforced the idea of pyrophosphate as a primordial energy currency (Baltscheffsky et al., 1966). Membrane-bound PPases couple the reversable phosphorylation of orthophosphate to pyrophosphate with the translocation of Na^+^ ions across a membrane. Pyrophosphate-dependent chemiosmosis appeared to align with one of the prevailing theories surrounding the origin of life; that hydrothermal environments provided geochemical chemiosmotic gradients of H^+^ or Na^+^ ions able to power primordial cells (Sleep et al., 2011; Lane and Martin, 2012; Martin et al., 2014; Lane, 2017). As cells evolved, originally porous membranes probably became less permeable and the energetic generation of ionic gradients was required, firstly using Na^+^ ions and later H^+^ ions (Mulkidjanian et al., 2008a,b). The prevalence of sodium ion gradients in extremophilic organisms and the promiscuity of many ATP synthases for both H^+^ and Na^+^ ions might suggest that the generation of Na^+^ ion gradients preceded H^+^ ion gradients (Lane and Martin, 2012). However, just like ATP synthases, membrane-bound PPases are large and complex enzymes unlikely to represent primordial bioenergetic systems (Martin and Thauer, 2017).

Pyrophosphate does play key roles in certain bioenergetic systems and the hydrolysis of pyrophosphate and its phosphoanhydride bond is highly exergonic, potentially indicating an ancient energetic role for this compound. Some prokaryotes have been found to hydrolyze pyrophosphate for energy in central metabolic reactions, for example one bacterium requires pyrophosphate during ATP production in the Wood-Ljungdahl pathway (James et al., 2016), and a pyrophosphate-dependent glycolytic pathway is present in many plants and some extremophilic microbes (Heinonen, 2001; Bielen et al., 2010). However, most metabolic reactions involving pyrophosphate merely produce it as a by-product from the hydrolysis of phosphorylated compounds. Relatively few metabolic reactions appear to require pyrophosphate to drive energetically unfavorable reactions and those that do might have adapted to specific ecological niches. Furthermore, investigations into the ancient metabolic pathways central to modern metabolism, and bioinformatic reconstructions of LUCA’s metabolism, have found no pyrophosphate consuming reactions or enzymes, suggesting that pyrophosphate was not utilized energetically by ancient organisms (Weiss et al., 2016; Coleman et al., 2021; Wimmer et al., 2021a).

While PPases are now known to be ubiquitous across all life and either soluble or membrane bound (Kajander et al., 2013), it appears they mainly function not to produce or conserve energy at all. With membrane bound PPases, an excess by-product is utilized in the energetically costly process of generating an ionic gradient. Notably, the most common PPases are cytosolic and appear not to utilize pyrophosphate energetically at all, acting only to produce orthophosphate. As such, pyrophosphate’s main cellular function is likely as a kinetic driver of ATP-consuming reactions, pushing organisms towards growth and further consumption of ATP (Wimmer et al., 2021a). With the ubiquitous PPases not representing precursors of ATP-based chemiosmosis and ATP synthases, but acting to remove pyrophosphate from driving the reverse of ATP-consuming reactions (Kornberg, 1962). Thus, the bioenergetic roles of pyrophosphate appear more likely the result of the evolution and expansion of ATP-based energy metabolism or recent adaptations to extreme environments, rather than the vestiges of ancient metabolism.

The main function of pyrophosphate as a metabolic by-product suggests that previously documented use of pyrophosphate as an environmental source of biological energy (Peck et al., 1983) is not due to its use as an energy currency, but more likely as a source of phosphate for ATP-based metabolism. For primordial cells, however, pyrophosphate could still represent a lucrative source of energy if present. Pyrophosphate is rare on the Earth today, being generally produced *via* intense heating of phosphate minerals in volcanic environments (Keefe and Miller, 1995). However, reduced phosphorus species that were likely common on the ancient Earth may have produced pyrophosphate under relevant conditions (Pasek, 2017). This could have been either from the dissolution of phosphide minerals such as schreibersite, delivered to Earth *via* meteorites, or *via* the direct oxidation of phosphite and hypophosphite (Bryant et al., 2013; Kee et al., 2013; Omran et al., 2022). Yet, even accounting for the increased volcanic activity and likely prevalence of reduced phosphorus species on the ancient Earth, the more reducing environment of the early Earth, coupled with the instability of pyrophosphate in aqueous solutions and its rarity in modern environments, suggests that pyrophosphate was unlikely to be abundant and available for early life in most scenarios (Schwartz, 2006).

### 4.2. Polyphosphate

Polyphosphates are inorganic, linear polymers consisting of three to hundreds of orthophosphate residues linked *via* high-energy phosphoanhydride bonds (Figure 3B). All extant cells utilize polyphosphate for the intracellular storage of phosphorus and energy within electron dense storage granules (Seufferheld et al., 2011; Lander et al., 2016) or, in some bacteria and eukarya, within a conserved and ancient organelle called the acidocalcisome (Frank et al., 2020; Greening and Lithgow, 2020). In addition to energy and phosphorus storage, the cellular roles of polyphosphate are extensive and include mediating stress responses, biofilm formation, virulence, gene regulation and metabolism (Rao et al., 2009; Muller et al., 2019). Polyphosphates are also important biochemical phosphorylating agents and may have played essential roles in the origin of life, phosphorylating essential biomolecules required in a heterotrophic scenario, or within early life itself (Schwartz, 2006; Guo et al., 2023). Polyphosphates can also function as a biochemical chaperone, potentially providing protection to proteins against numerous cellular stresses before the advent of chaperone proteins (Gray et al., 2014). The widespread, diverse, and conserved cellular functions of polyphosphates across all domains of life highlight their ancient origins in biochemistry.

The well-established and ancient role of polyphosphate as a cellular store of energy has led many to suggest that it may also have acted as a primordial energy currency, along with its chemical similarities with the triphosphate moiety of ATP. Polyphosphate is also able to substitute for ATP in a variety of metabolic reactions and some enzymes utilizing ATP as substrate are also able to use polyphosphate (Kornberg et al., 1999; Albi and Serrano, 2016). Additionally, polyphosphate is intrinsically linked to ATP in modern metabolism through its synthesis and degradation. The major prokaryotic enzymes involved in polyphosphate metabolism are widely conserved across many bacteria, archaea, and some single-celled eukaryotes (Wang et al., 2018; Muller et al., 2019; Paula et al., 2019; Wang et al., 2019). Polyphosphate kinase 1 (PPK1) is responsible for the majority of intracellular synthesis of polyphosphate via the transfer of phosphate from ATP, while polyphosphate kinase 2 (PPK2) has a general preference for polyphosphate degradation coupled to phosphorylation of ADP or GDP. Some bacteria and archaea also possess exopolyphosphatase (PPX) enzymes that processively cleave polyphosphate and release inorganic phosphate, although the question of where that energy goes remains unknown. Many prokaryotes also lack characterized polyphosphate metabolism machinery, highlighting the potential for polyphosphate to play further currently unrecognized roles in energy metabolism (Wang et al., 2019). The presence of polyphosphate metabolism machinery in LUCA is unknown but the conservation of many genes across bacteria and archaea suggests an ancient origin of polyphosphate metabolism. The role of polyphosphate in an important ATP synthesis mechanism may also imply polyphosphate could have been utilized energetically prior to ATP.

Possessing sometimes hundreds of highly energetic phosphoanhydride bonds has also led to the suggestion that polyphosphate could be an energetically rich source of environmental energy for ancient life (Kornberg, 1995; Achbergerová and Nahálka, 2011). Today, certain marine microorganisms are able to consume environmental polyphosphates as their sole source of phosphorus for growth and may even prefer polyphosphates over typical sources of biological phosphorus, although it remains unknown how widespread this recently discovered metabolic trait is (Diaz et al., 2018, 2019; Adams et al., 2022). It is hypothesized that microbes could be utilizing polyphosphates as an environmental source of energy, not just phosphorus, due to the catabolism of polyphosphate under non-limited phosphorus conditions (Peck et al., 1983; Filella et al., 2022). It has also been suggested that the prokaryotic PPK1 enzyme evolved after PPK2, indicating that degradation of polyphosphates could be older than synthesis. Thus, supporting the idea that polyphosphate could have acted as an ancient source of energy and phosphorus, with organisms later evolving synthesis machinery after polyphosphates became environmentally limited (Achbergerová and Nahálka, 2014).

Polyphosphates are produced synthetically relatively easily from the intense heating of various phosphate salts and minerals, in a similar mechanism to the related pyrophosphates. Also similarly to pyrophosphates is their rarity on the current Earth (Keefe and Miller, 1995), having only been discovered in a single calcium mineral deposit (Rouse et al., 1988) or as short chain polymers forming within volcanic fumaroles (Yamagata et al., 1991). A lack of a significant abiotic source for polyphosphate makes it an unlikely candidate to have sustained early life (Keefe and Miller, 1995). Although the biological consumption of environmental polyphosphate could be an ancient metabolic trait that resulted in most primordially produced polyphosphate being depleted, it may also represent a recent adaptation to the widespread biological synthesis of polyphosphate by modern organisms. However, the crucial and multifaceted roles of polyphosphate in modern metabolism, especially including its hypothesized use as an external source of biological energy, clearly point to its ancient and potentially pre-ATP origins. The diversity and evolution of polyphosphate metabolism is likely intricately linked with the evolution of energy metabolism.

### 4.3. Acetyl phosphate

Acetyl phosphate is a simple organophosphate molecule with a highly energetic phosphoester bond (Figure 3C) that shares similarities with a class of compounds called thioesters that are heavily implicated in the origins of life and bioenergetics. Thioesters are organosulfur compounds in which the phosphate of acetyl-phosphate is replaced by a thiol group. Thioesters, such as acetyl coenzyme A (acetyl CoA), are ubiquitous metabolic intermediates in modern energy metabolism (i.e., the TCA cycle) and are precursors to acetyl phosphate synthesis. Acetyl CoA and acetyl phosphate are also both central in the chemoautotrophic Wood-Ljungdahl pathway, often suggested to be the most ancient of the extant carbon fixation pathways and a vestige of the earliest bioenergetic systems (Martin, 2020).

The Wood-Ljungdahl pathway is conserved across many extremophilic bacteria and archaea, along with being inferred in LUCA. The pathway is centered around enzymes containing iron–sulfur (FeS) cluster cofactors, such as ferredoxins, which catalyze reactions involving metabolic intermediates primed by energy-dense thioester linkages. ATP is produced consequently *via* SLP, with CO_2_ being fixed *via* the oxidation of molecular hydrogen (H_2_). The central thioester metabolites of the Wood-Ljungdahl pathway are suggested to have been involved in the synthesis of essential biomolecules at the origin of life. While acetyl CoA is a complex molecule requiring ATP for synthesis in modern metabolism, several abiotic processes might have produced simpler thioester compounds that could have been the basis for what would evolve into the Wood-Ljungdahl pathway (Fuchs, 2011; Martin, 2020; Preiner et al., 2020). It is further suggested that the CO_2_ and H_2_ redox couple utilized in the Wood-Ljungdahl pathway could also have provided the earliest source of energy for primordial autotrophic organisms (Russell et al., 2010; Preiner et al., 2018; Sousa et al., 2018), with the fixation of CO_2_ also being catalyzed by primordial FeS proto-enzymes or minerals (Wächtershäuser, 2006; Herschy et al., 2014; Goldford et al., 2019). Serpentinization in hydrothermal environments, such as alkaline hydrothermal vents, in the early oceans would have likely provided the iron, sulfur, H_2_ and CO_2_ necessary for such processes (Sleep et al., 2011; Camprubi et al., 2017; Mutter et al., 2019).

Simple thioester metabolites may have also been the basis of early metabolic networks in a primordial ‘thioester world’ prior to the evolution of ATP-based metabolism (De Duve, 1995; Hartman and Smith, 2019; Frenkel-Pinter et al., 2022). Likely abundant minerals of iron and sulfur might have acted as the first proto-enzymes, providing an environmental source of energy without phosphate and phosphorylated nucleotides. Instead, simple abiotically produced organosulfur compounds containing energy-dense thioester bonds might have been utilized in a heterotrophic origin of energy metabolism. The formation of primordial FeS cluster enzymes from minerals might have even occurred by UV photo-oxidation on the early Earth (Bonfio et al., 2017). A hypothetical thioester metabolism utilizing prebiotic precursors has been shown to synthesize key organic compounds and exhibit crucial functions of a metabolic network (Semenov et al., 2016). Additionally, analysis of universally conserved metabolic networks in extant organisms has found the potential remnants of a simplified metabolic network able to support all major metabolic functions based solely around thioester-linked metabolites and iron–sulfur cluster enzymes (Goldford et al., 2017; Martin and Thauer, 2017). A primordial thioester-based metabolism catalyzed by FeS minerals or proto-enzymes might also have been able to synthesize phosphorylated biomolecules including ATP, providing a potential evolutionary link between an earlier thioester world and the modern phosphate world (Hartman and Smith, 2019).

The potential for a primordial FeS and thioester-based metabolism to have evolved into ATP-based metabolism might place acetyl phosphate, involved in both, in a key role in the evolution of energy metabolism. Acetyl phosphate has been demonstrated to be interchangeable with ATP in certain biochemical reactions (Whicher et al., 2018) and, along with being heavily involved in the Wood-Ljungdahl pathway as an intermediate in the generation of ATP from acetyl thioesters, could illustrate the direct evolution of energy metabolism from a thioester world (Martin and Thauer, 2017). Moreover, acetyl phosphate might also have formed abiotically from simple methyl thioesters likely prevalent on the prebiotic world, potentially acting as a prebiotic phosphorylation agent and source of environmental energy for primordial life (De Duve, 1995). Acetyl phosphate can be relatively easily synthesized in water under mild conditions from potentially prebiotic precursors and can also perform biologically relevant phosphorylations under these conditions (Whicher et al., 2018). However, acetyl phosphate is unable to promote polymerization of biomolecules (an essential metabolic process during the prebiotic origin of life) and some doubt whether the compound is reactive enough to have provided a prebiotic source of metabolic energy in the absence of efficient catalysis (Liu et al., 2019). Acetyl phosphates could thus be a relic of the thioester world, providing an evolutionary bridge from thioester metabolism into the modern, phosphate-based metabolic world.

## 5. Reduced phosphorus species in ancient energy metabolism

**Figure 4:**
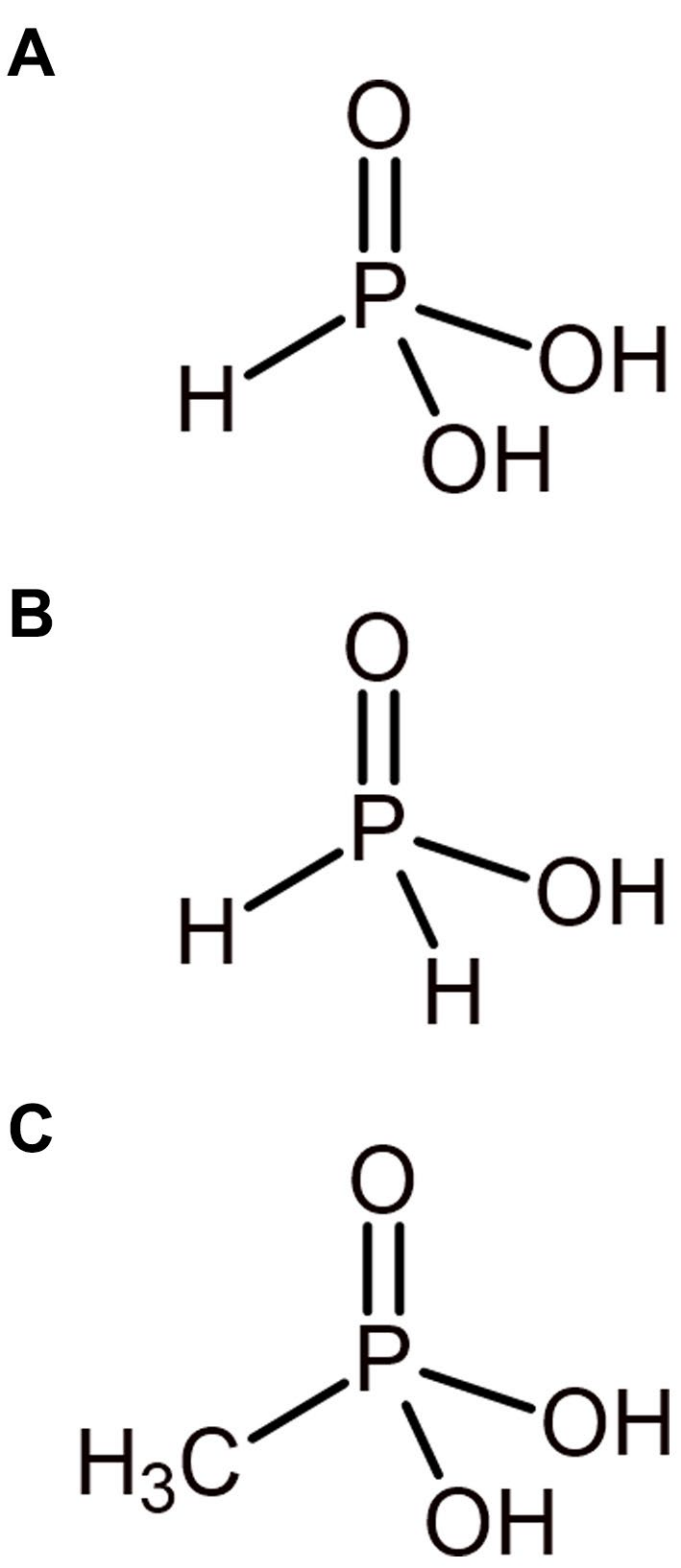
Primordially relevant reduced phosphorus compounds. Phosphorus exists in the reduced P^3+^ and P^1+^ oxidation state as phosphite **(A)** and hypophosphite **(B)** respectively. Methylphosphonate **(C)** is an example of a simple phosphonate, where phosphorus is in the P^3+^ oxidation state.

Phosphorus is often assumed to exist almost entirely as phosphate and mainly in the oxidized P^5+^ redox state on Earth today. However, phosphorus can also exist in the reduced P^3+^ and P^1+^ oxidation states as phosphite (PO_3_^3−^) and hypophosphite (PO_2_^3−^) respectively, alongside being incorporated into reduced organophosphonate compounds also in the P^3+^ oxidation state (Figure 4). A largely overlooked phosphorus redox cycle is beginning to emerge (Pasek, 2019; Liu et al., 2023), with these redox species of phosphorus being found in diverse environments on the current Earth and in environments potentially analogous to the ancient Earth, such as hydrothermal systems (Pech et al., 2009). The ancient Earth’s atmosphere and oceans may also have been able to sustain more reduced phosphorus species by being less oxidizing than today. Reduced phosphorus compounds are more available to biology than apatite minerals (Gulick, 1955) and, therefore, may be compelling sources of phosphorus for early life, circumventing the potential problem of orthophosphate sequestration on the early Earth (Pasek, 2008).

### 5.1. Organophosphonates

Organophosphonates contain a highly stable C – P bond and are utilized by diverse microorganisms today as alternative sources of phosphorus when experiencing phosphate starvation. Phosphonates make up a significant fraction of the microbial P cycle in modern environments and have long been considered ancient, and potentially prebiotic, molecules that might have provided early life with an alternative, abiotically produced source of phosphorus (Mcgrath et al., 2013). It appears likely that biological synthesis is the major input of phosphonates into modern environments (Villarreal-Chiu et al., 2012). However, phosphonates have also been detected as dissolution products of the likely prebiotically relevant phosphide meteorites mentioned earlier (Pasek and Lauretta, 2005) and could also have been delivered to the early Earth *via* carbonaceous meteorites (Cooper et al., 1992).

While many phosphonate compounds contain a phosphoryl group required for the phosphorylation of biomolecules or transfer of cellular energy in phosphorylated bioenergetic molecules, the strength of the C – P bond and the energy required to cleave it suggests that phosphonates are unlikely candidates for primordial bioenergetic molecules. Nevertheless, phosphonate catabolism could still represent a primordially relevant source of phosphorus, as illustrated by the observation of significantly upregulated microbial organophosphonate consumption within hydrothermal serpentinizing environments (Frouin et al., 2022). Modern microorganisms have developed specialized and complex biochemical pathways for the cleavage of organophosphonates, the most widespread of which is the C-P lyase pathway, consisting of many accessory genes and a large enzyme complex usually all encoded by one gene operon. The evolution of C-P lyase, while conserved across many diverse bacteria and archaea, appears to be mainly the result of lateral transfer (Huang et al., 2005) and, additionally, the pathway requires ATP at multiple steps. While other potentially simpler pathways and enzymes exist (Villarreal-Chiu et al., 2012) and might have primordial relevance, the complex and energetically costly catabolism of organophosphonates might suggest a more recent and niche metabolic adaptation occurring at least after the onset of recognizably modern energy metabolism.

### 5.2. Phosphite and hypophosphite

Phosphite is a reduced phosphorus species (PO_3_^3−^) that represents a hitherto largely ignored pool of soluble and reactive phosphorus that can be found in diverse modern environments (Hanrahan et al., 2005; Stone and White, 2012), and might have been widespread and available to primitive biology on the ancient Earth (Pasek, 2019). Notwithstanding the reducing atmosphere and oceans that might have favored and sustained reduced phosphorus species, numerous other sources of phosphite might have existed. Phosphates produced on the early Earth *via* volcanism or other aforementioned mechanisms may have been reduced to phosphite *via* lightning activity (Schwartz, 2006; Pasek and Block, 2009; Bindi et al., 2023) or the oxidation of iron at hydrothermal environments (Pech et al., 2009; Herschy et al., 2018). Schreibersite, as mentioned previously, is a phosphide mineral which might have heavily influenced the abundance and catalogue of bioavailable phosphorus on the early Earth by dissolving under various primordial conditions to produce, in addition to pyrophosphate and orthophosphate, phosphite and hypophosphite (Pasek and Lauretta, 2005; Bryant et al., 2013). The ability of phosphide minerals to produce reduced phosphorus molecules that would not be sequestered by minerals on the ancient Earth and the potential abundance of schreibersite on the early Earth suggests that phosphite could have played a largely underappreciated role in the evolution of early life as a phosphorylating agent and a potential source of energy *via* phosphite oxidation (Tapia-Torres and Olmedo-Álvarez, 2018).

The oxidation of phosphite to phosphate is associated with an energy release of −77 to −91 kJ/mol, making it a potentially lucrative source of environmental energy for early life and the most energetically favorable of all known chemotrophic electron donors (Poehlein et al., 2013). On the Earth today, a diverse range of bacteria, including *Escherichia coli*, are able to utilize phosphite and hypophosphite for assimilation into biomass, *via* certain promiscuous C-P lyase and alkaline phosphatase enzymes (Casida, 1960; Foster et al., 1978; Metcalf and Wanner, 1991). While first discovered in the 1960’s, the assimilatory oxidation of phosphite and hypophosphite (known as assimilatory phosphite oxidation, APO) has only recently begun to be characterized (Metcalf and Wolfe, 1998; White and Metcalf, 2007) and understood as playing a role in a previously unrecognized global biogeochemical phosphorus redox cycle (Pasek, 2019). Aside from C-P lyase and phosphatases, one other highly specific enzyme has been shown to oxidize phosphite, phosphite dehydrogenase (PtxD). Encoded for as part of the *ptxABCDE* gene operon, PtxD catalyzes the assimilation of phosphite into microbial biomass along with a phosphite membrane transporter (PtxABC) and a transcriptional regulator (PtxE) (White and Metcalf, 2004b). The *ptx* operon is also often found along with a hypophosphite oxidizing C-P lyase homolog, representing a direct route to the assimilation of phosphate from hypophosphite.

However, PtxD can also function to utilize phosphite as the sole electron donor, coupling the reduction of carbon dioxide, sulfate or nitrate to the oxidation of phosphite in dissimilatory phosphite oxidation (DPO) (Schink and Friedrich, 2000). The oxidation of phosphite as a source of electrons and energy was first identified in 2000 by Schink et al., who discovered a chemoautotrophic bacterial strain called *Desulfotignum phosphitoxidans* able to grow on phosphite as its sole energy source. There are currently only two other characterized and isolated organisms known to perform DPO, along with several others identified from metagenomic analysis of environmental samples (Figueroa et al., 2018; Ewens et al., 2021; Mao et al., 2021). It remains unknown what exactly confers the ability of an organism and its PtxD enzyme to perform DPO instead of APO (Simeonova et al., 2010). The APO and DPO pathways are usually distinguished by their unique and usually conserved gene clusters, with DPO organisms specifically containing the *ptxDE-ptdCFGHI* gene cluster (White and Metcalf, 2004a; Ewens et al., 2021).

DPO is suggested to be an ancient metabolic trait, with organisms able to perform DPO being strictly anaerobic and fixing carbon *via* the likely ancient Wood-Ljungdahl pathway (Poehlein et al., 2013). Additionally, the *ptx-ptd* gene clusters of discovered DPO organisms appear to show vertical inheritance and, along with the taxonomic diversity of these organisms, may point to an ancient origin of this gene cluster (Ewens et al., 2021). Homologs of *ptxD* are also found across bacteria and archaea, supporting an ancient origin (Figueroa and Coates, 2017), yet it remains unknown whether homologs might confer the APO or DPO trait. The *ptxD* gene also shares significant homology with many other members of its broader enzyme class, the D-2-hydroxyacid dehydrogenases, which makes it unclear whether these homologs may be functionally similar. Additionally, knowing only several DPO capable organisms makes it hard to draw conclusions on this trait’s evolutionary history, especially given that most organisms identified to date belong to a single phylum of extremophilic bacteria, the *Thermodesulfobacteriota.* The potential for DPO to have spread *via* horizontal gene transfer within ecological niches, likely how *D. phosphitoxidans* acquired its DPO gene cluster, should also not be discounted (Simeonova et al., 2010; Figueroa and Coates, 2017).

The diversity and distribution of phosphite oxidation remains uncertain, with the diversity of DPO even more so due to the ambiguity surrounding the exact mechanisms of PtxD catalysis and the requirements of DPO compared to APO. The apparent lack of archaeal DPO organisms and the small number of bacterial ones raises questions over whether this trait could have been ancient and pre-LUCA. However, this could be explained by sampling and culturing limitations or simply be the result of gene loss over time. The microbial production of phosphite *via* phosphate reduction has also been suggested (Pasek et al., 2014) which, if coupled with phosphite utilization, suggests an intriguing role for phosphite as an ancient energy currency. However, the energy barrier to reduce phosphate to phosphite is significant, not ideal in a potential energy currency, and the mechanisms by which microbial phosphate reduction may occur remain unknown (White and Metcalf, 2007). It seems clearer that the ability to assimilate and oxidize phosphite for biomass is widespread within modern prokaryotes and, therefore, it might seem plausible that the energetic pathway could have been lost over time due to environmental changes. The possibility of a significant phosphorus redox cycle on the early Earth, coupled with the energetic and biological potential of phosphite, suggests that phosphite may still have played an important role in early evolution, and elucidating the antiquity and diversity of modern microbial phosphite oxidation pathways will likely be crucial in determining this role.

## 6. Discussion

Phosphorus undoubtedly played an essential role in the early evolution of modern life as a component of DNA, RNA, and the universal energy currency, ATP. However, understanding how phosphorus was first incorporated into biomolecules, and specifically energy metabolism, during pre-LUCA evolution, and potentially back to the origin of life, remains unclear. While evolution can occur rapidly compared to geological timescales, and ancient enzymes are often found to be highly adapted and evolved (Kędzior et al., 2022), the onset of energy metabolism was surely simpler than ATP synthases coupled to chemiosmotic gradients and substrate-level phosphorylation *via* ancestral carbon fixation pathways.

It is conceivable that ATP may have been selected as the universal energy currency during an evolutionary event occurring at or soon after the origin of life, anchoring the molecule intrinsically to biological energy metabolism from the outset. Primordial life scavenging phosphate from alternative environmental sources, including from the alternative phosphate-containing compounds discussed here, could have synthesized phosphorylated energy currencies, including ATP, through simpler, proto-metabolic systems. There also exists the possibility that phosphorylated nucleotides were being produced abiotically on the ancient Earth (Pasek et al., 2017; Chu and Zhang, 2023; Guo et al., 2023), thereafter providing the energetic impetus for life during a potentially heterotrophic origin. However, if any such alternative ATP-based metabolic systems existed that did not require chemiosmotic ATP synthases or SLP pathways, it would appear that no such ancestors of these systems remain today. The abiotic synthesis of phosphorylated nucleotides is also generally considered complex, predicated on the existence of niche environments and non-aquatic solvents that may or may not have been present on the early Earth (Chu and Zhang, 2023). Therefore, the possibility of precursors to ATP existing in primordial biochemistry appears worth considering and may help solve the coupled problems of primordial energy metabolism on a phosphate limited ancient Earth.

**Figure 5:**
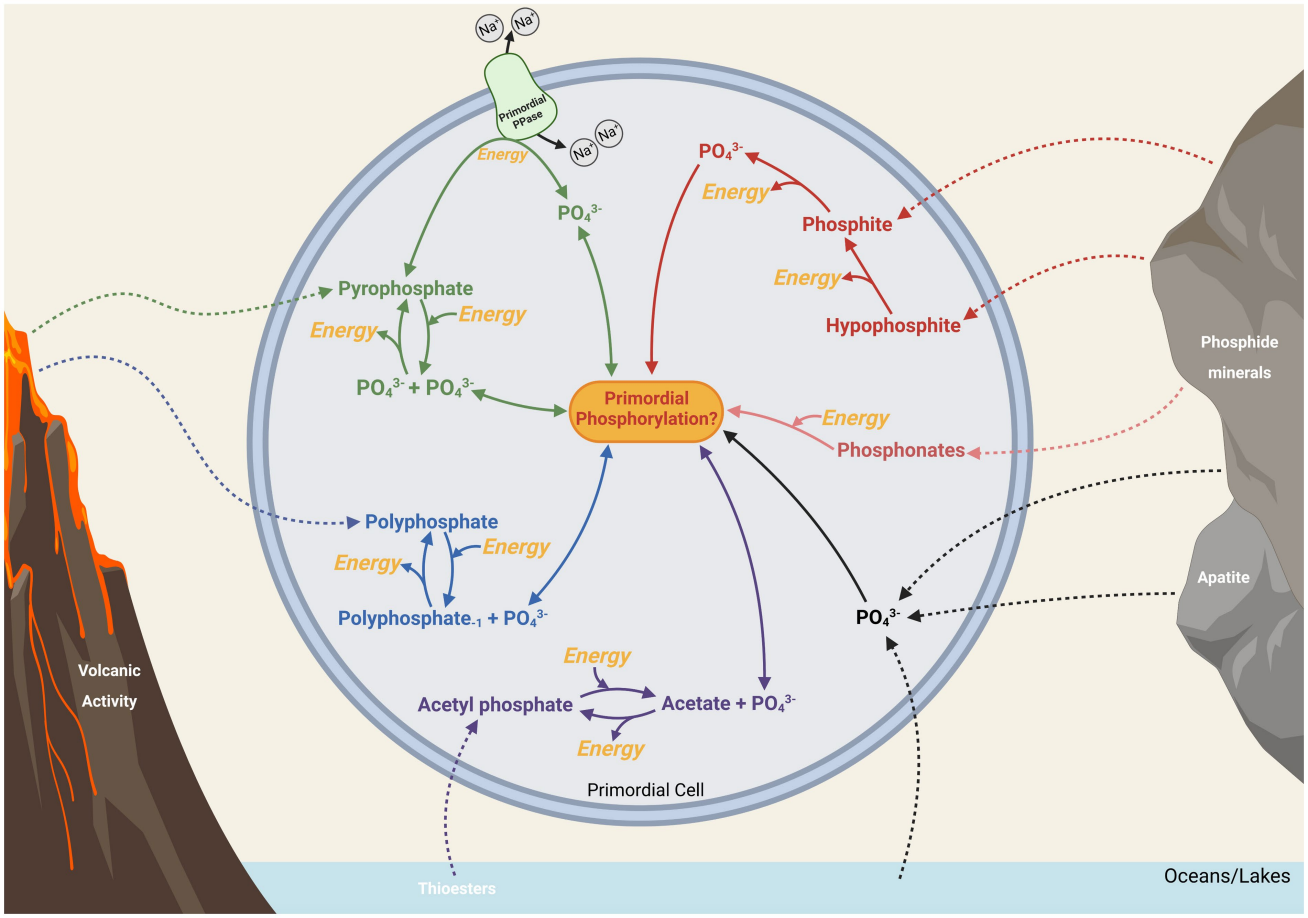
Potentially primordial routes of intracellular and extracellular phosphate (PO_4_^3−^) that might have been available to biology for metabolic phosphorylation and energy. Energetically costly and beneficial processes are highlighted in yellow. Possible major sources of phosphorus from environmental sources are shown with dashed lines (see Figure 2 for a more detailed representation), while potential intracellular pathways are solid lines.

It is illustrated here how the compounds pyrophosphate, polyphosphates, and acetyl phosphate play integral roles in extant energy metabolism due to their highly energetic bonds and importance as metabolic intermediates. While pyrophosphate mainly appears as a metabolic by-product in energy metabolism, the ubiquity of polyphosphate in energetic pathways, along with its continual synthesis and degradation within cells, may point to a role for this biopolymer as an ancient energy currency. Acetyl phosphate, being central in the ancient Wood-Ljungdahl pathway, may have particular relevance in the early evolution of that pathway, potentially providing a link between phosphate-based metabolism and a primordial proto-metabolism based around thioesters and FeS cluster minerals or proto-enzymes. Additionally, abiotically produced acetyl phosphate, pyrophosphate and polyphosphates might have provided energy for proto-biochemistry in a heterotrophic origin of life scenario. These compounds may also have acted as environmental sources of phosphorus and energy after primordial life evolved, assuming mechanisms existed to transfer these compounds across early membranes (see Figure 5).

Phosphorylated compounds were unlikely to be widespread on the ancient Earth due to thermodynamic instabilities and the potentially limiting factor of phosphate availability. However, there now exists several plausible theories explaining how primordial life could have attained phosphate - either through the dissolution of minerals and meteorites (including reduced phosphide minerals) or solubilized in niche geological and aquatic environments. As such, primordial life may have been able to attain bioavailable phosphate to synthesize phosphorylated energy currencies and other essential biomolecules. Nonetheless, such environments may have remained scarce, with free phosphate likely sequestered by calcium and iron minerals to some extent.

Reduced phosphorus compounds, in addition to providing potentially significant bioavailable phosphorus in the form of phosphide minerals, could have provided the energetic impetus for early life and the evolution of a phosphate-based biochemical world (Figure 5). The apparent abundance of phosphite on the early Earth and its use as an energy source in some modern extremophiles provides an enticing solution to the question of how primordial life scavenged energy and/or phosphorus from a world possibly limited in phosphate. However, the evolution and diversity of microbial phosphite oxidation mechanisms remains largely unknown. Further investigation of this and other understudied metabolic traits including the biological utilization of polyphosphates and pyrophosphates will likely provide invaluable insights into these potentially ancient metabolic traits.

There remain significant challenges in combining the approaches of molecular biology and phylogenetics to infer the biochemistry of our earliest ancestors, with the biogeochemical approaches attempting to recreate the conditions surrounding the early evolution of life. While this review has attempted to consolidate and compare our current understanding of these subjects relating to energy metabolism, there are still significant gaps in our understanding of the evolutionary steps involved in metabolism from life’s origin to the existence of LUCA. The advance of phylogenetics and metabolic modelling will likely continue to provide clearer insights into the metabolism of our ancient ancestors, while planetary and geochemical science continues to improve our understanding of the conditions surrounding the origins and early evolution of life. It is hoped that further study into the diverse bioenergetic pathways involving the alternative phosphorus compounds highlighted here might help elucidate the evolution of primordial metabolism, providing insights into integral stages in the evolution of life on Earth and potentially other planets.

## Author contributions

JN prepared the manuscript and figures. JC, TW, TL, VO’F, and JM contributed to, edited, and reviewed the manuscript. All authors contributed to the article and approved the submitted version.

## Funding

This work was supported by a studentship from the Northern Irish Department for the Economy and a grant from the Biotechnology and Biological Sciences Research Council (BB/W019531/1).

## Conflict of interest

The authors declare that the research was conducted in the absence of any commercial or financial relationships that could be construed as a potential conflict of interest.

## Publisher’s note

All claims expressed in this article are solely those of the authors and do not necessarily represent those of their affiliated organizations, or those of the publisher, the editors and the reviewers. Any product that may be evaluated in this article, or claim that may be made by its manufacturer, is not guaranteed or endorsed by the publisher.
